# Impact of elevated body mass index on cumulative live birth rate and obstetric safety in women undergoing assisted reproductive technology

**DOI:** 10.1038/s41598-022-23576-0

**Published:** 2022-11-07

**Authors:** Dan Hu, Bo Huang, Min Xiong, Junning Yao, Shulin Yang, Ruxing Wu, Hanwang Zhang, Yiqing Zhao

**Affiliations:** Reproductive Medicine Center, Tongji Hospital, Tongji Medical College, Huazhong University of Science and Technology, 1095 JieFang Avenue, Wuhan, 430030 People’s Republic of China

**Keywords:** Obesity, Infertility

## Abstract

This study evaluated the impact of elevated body mass index (BMI) on short- and long-term outcomes of in-vitro fertilization (IVF)/intracytoplasmic sperm injection (ICSI) treatments. A total of 7229 patients undergoing IVF/ICSI fresh cycles and subsequent frozen embryo transfer cycles from 2014 to 2020 were divided into normal (18.5–24.9 kg/m^2^) and high BMI (≥ 25 kg/m^2^) groups. Ovarian response, pregnancy outcomes, and safety of both mother and fetus were the main outcome measures. Furthermore, multivariate analysis was used to determine whether BMI was associated with cumulative live birth rate (CLBR). Results showed that for younger women (< 38 year), CLBR was significantly reduced in the high BMI group compared with the normal BMI control and was accompanied by fewer retrieved oocytes and available embryos. Additionally, the incidence of hypertensive disorders of pregnancy, fetal macrosomia, and cleft lip and palate birth defects resulting from cumulative live births was significantly higher compared with the normal BMI group. No differences were observed among older women (≥ 38 year). Multivariate analysis revealed that high BMI was a risk factor for CLBR. Our study suggested that elevated BMI has a greater adverse impact on younger women.

## Introduction

The overweight and obesity epidemic continues to plague modern society, which is a result of radical changes to lifestyle and decreased physical exercise in recent years. Overweight and obesity are major causes of chronic diseases, such as cardiovascular disease and type 2 diabetes^1–3^. Moreover, obesity raises the risk of hypertension and stroke, dyslipidemia, diabetes, coronary heart disease, as well as various forms of cancer. Obese people are also three times more likely to be hospitalized by COVID-19^4^.

The burden of overweight and obesity is high in both developed and developing countries, with the proportion of overweight and obese women increasing from 29.8% in 1980 to 38.0% in 2013^5^. Elevated body mass index (BMI) in women affects every stage of reproductive life, including puberty, pregnancy, and delivery. This may include conditions such as menstrual and ovulatory disorders, impaired endometrial development and embryo implantation, increased abortion rates, and pregnancy complications, such as hypertensive disorders of pregnancy (HDP), pre-eclampsia, gestational diabetes mellitus (GDM), postpartum hemorrhage (PPH), and cesarean delivery^6^. Moreover, adverse perinatal outcomes, including fetal macrosomia and neural tube defects, are more likely to occur in obese women^7^.

Numerous studies investigating the impact of elevated BMI on in-vitro fertilization (IVF)/intracytoplasmic sperm injection (ICSI) outcomes have been published, albeit with disparate results. Several studies found that an elevated BMI had no adverse effect on IVF/ICSI outcomes^8–10^, while others demonstrated that elevated BMI was associated with adverse IVF outcomes, including lower ovarian response, inferior oocyte and embryo quality, higher cancellation rates, lower clinical pregnancy rates (CPR), lower fresh live birth rates (FLBR), and higher rates of miscarriages^11–18^. Despite broad research in this area, the effects of elevated BMI on IVF/ICSI outcomes remain uncertain.

Therefore, this study aimed to evaluate the short-term effects of elevated BMI on IVF/ICSI, primarily on ovarian response, embryo quality, pregnancy outcome of fresh cycle. In addition, long-term effects were evaluated, including obstetric complications, perinatal outcomes, and congenital defects associated with deliveries from fresh and cumulative cycles. We aimed to elucidate the impact of high BMI on IVF/ICSI more comprehensively and potentially inform pre-IVF/ICSI counseling of overweight and obese women. Since a woman’s age is the single most important determinant of fertility, the following factors were considered in this study when patients aged below 38 years and aged 38 years and above were studied separately: Firstly, the total number of oocytes gradually decreases with age, with a sharp decline at the age of 37.5^19–22^, and cumulative live birth rate (CLBR) significantly decreases with the decrease number of retrieved oocytes^23^. Second, oocyte and embryo competence decrease with maternal age, mainly due to an increased incidence of aneuploidies and possibly decreased mitochondrial activity^24^, with up to 70–80% of embryos from females aged 38–42 demonstrating chromosomal abnormalities^25,26^. Third, potential cardiovascular risk^2,3^, adverse obstetrical and perinatal outcomes also increase with advanced maternal age^27^.

## Results

### Patient characteristics

Of the 7229 women who completed the follow up, 5404 achieved at least one live birth in the first stimulation and 1825 did not achieve a live birth after using up all embryos (Supplementary Fig. S1). Moreover, the 7229 participants were divided into two BMI groups—normal BMI and high BMI. Baseline characteristics of the participants aged below 38 years are summarized in Table 1. Overall, 5881 women had normal weight (BMI = 21.28 ± 1.71), while 977 women were considered overweight and obese (BMI = 26.80 ± 1.74). No significant differences in the type and etiology of infertility, and fertilization method between the two BMI groups were noted. Basal follicle-stimulating hormone (FSH) (6.6 vs. 6.89, Z = -5.350, *P* < 0.001) and antral follicle count (AFC) (15 vs. 14, Z = 2.383, *P* = 0.017) were significantly lower and higher, respectively, in the high BMI group compared with the normal BMI group. In addition, baseline characteristics of the participants aged 38 years and above are summarized in Supplementary Table S1. Overall, 293 women had normal weight (BMI = 22.03 ± 1.62), while 78 women were considered overweight and obese (BMI = 27.12 ± 2.00). No significant differences in baseline characteristics between the two BMI groups were noted.

**Table 1:** Comparison of baseline characteristics among normal and high BMI^a^ groups (< 38 year).

	Normal BMI^a^	High BMI^a^	χ^2^/z	*P* value
**Infertility type**			2.462	0.117
Primary	3420 (58.2%)	542 (55.5%)		
Secondary	2461 (41.8%)	435 (44.5%)		
**Infertility etiology**			9.038	0.060
Male	2015 (34.3%)	329 (33.7%)		
Tubal	3363 (57.2%)	575 (58.9%)		
DOR^b^	42 (0.7%)	12 (1.2%)		
EMT^c^	217 (3.7%)	21 (2.1%)		
Unexplained infertility	244 (4.1%)	40 (4.1%)		
**Fertilization method**			0.206	0.650
IVF^d^	4196 (71.3%)	704 (72.1%)		
ICSI^e^	1685 (28.7%)	273 (27.9%)		
Duration of infertility (years)	3 (2–5)	3 (2–5)	5.148	< 0.001*
Basal FSH^f^ (IU/L)	6.89 (5.93–7.92)	6.6 (5.63–7.57)	− 5.350	< 0.001*
AFC^g^ (n)	14 (11–19)	15 (11–21)	2.383	0.017*

^a^Body mass index.

^b^Diminished ovarian reserve.

^c^Endometriosis.

^d^In-vitro fertilization.

^e^Intracytoplasmic sperm injection.

^f^Follicle-stimulating hormone.

^g^Antral follicle count.

*P < 0.05.

### Parameters of ovarian stimulation and embryos

Comparisons of the parameters for ovarian response between the two BMI categories are presented in Table 2 and Supplementary Table S2. Younger women (< 38 y) with high BMIs received a significantly higher dose of gonadotropin (Gn) stimulation (2175 vs. 1960, z = 10.255, *P* < 0.001) and had a slightly longer duration of Gn stimulation (10 vs. 10, z = 1.975, *P* = 0.048) than those with normal BMIs. Fewer retrieved oocytes (13 vs. 14, z =− 3.312, *P* < 0.001), metaphase II oocytes (12 vs. 12, z = -3.322, *P* < 0.001), normally fertilized oocytes (8 vs. 8, z = -3.324, *P* < 0.001), cleavages (9 vs. 10, z = − 3.816, *P* < 0.001), cleavage-stage embryos (8 vs 8, z = − 2.978, *P* = 0.003), and blastocysts (4 vs 4, z = − 2.621, *P* = 0.009) were noted in the high BMI group (Table 2). However, in the older women (≥ 38 y) subgroup, the duration of Gn stimulation was shorter (10 vs. 10, z = − 2.802, *P* = 0.005) and the cleavage was lower (7 vs 7, z = − 2.141, *P* = 0.032) in the high BMI group compared with the normal BMI group (Supplementary Table S2). In both age subgroups, serum peak estradiol and progesterone (P) concentrations on human chorionic gonadotropin (hCG) day were significantly lower in the high BMI group compared with the normal BMI group. No significant differences in the numbers of high-quality embryos (HQE) and top-quality embryos (TQE) were observed between the two BMI groups (Table 2 and Supplementary Table S2).

**Table 2:** Parameters of ovarian stimulation and embryos (< 38 year).

< 38 year	Normal BMI^a^	High BMI^a^	z	*P* value
Duration of Gn^b^ (days)	10 (9–11)	10 (9–11)	1.975	0.048*
Total Gn^b^ dose (IU)	1960 (1537.5–2385)	2175 (1770–2587.5)	10.255	< 0.001*
Peak estradiol (pg/ml)	4276 (3000–6038)	3470 (2464–5013)	− 10.421	< 0.001*
Serum P^c^ on hCG^d^ day (ng/ml)	1 (0.77–1.26)	0.9 (0.7–1.16)	− 7.109	< 0.001*
Endometrium thickness (mm)	11.5 (10–13.1)	11.6 (9.9–13.2)	0.553	0.580
No. of follicles ≥ 14 mm (n)	11 (9–14)	11 (9–14)	0.766	0.444
Retrieved oocytes (n)	14 (10–18)	13 (9–17)	− 3.312	< 0.001*
MII oocytes (n)	12 (9–16)	12 (8–15)	− 3.322	< 0.001*
MII oocytes rate (%)	92.31 (84.21–100)	92.59 (83.33–100)	0.691	0.489
**Insemination procedure (%)**				
IVF^e^	63.64 (50–75)	62.5 (50–75)	− 0.797	0.426
ICSI^f^	73.68 (61.54–83.33)	73.33 (61.54–84.62)	− 0.258	0.796
Normally fertilized oocytes (n)	8 (6–11)	8 (5–10)	− 3.324	< 0.001*
**Fertilization rate (%)**				
IVF^e^	63.64 (50–75)	62.5 (50–75)	− 0.503	0.615
ICSI^f^	73.68 (61.54–83.33)	73.33 (60–84.62)	− 0.140	0.888
Cleavage (n)	10 (7–13)	9 (6–13)	− 3.816	< 0.001*
Cleavage rate (%)	100 (100–100)	100 (100–100)	0.974	0.330
Cleavage-stage embryos (n)	8 (6–11)	8 (6–11)	− 2.978	0.003*
Cleavage-stage embryos rate (%)	84.62 (72.73–100)	85.71 (75–100)	1.755	0.079
Blastocyst (n)	4 (2–7)	4 (2–6)	− 2.621	0.009*
Blastocyst formation rate (%)	62.5 (40–81.25)	60 (40–81.82)	− 0.705	0.481
Available blastocyst rate (%)	37.5 (16.67–55.56)	37.5 (14.29–55.56)	− 0.511	0.610
HQE^g^ (n)	4 (2–6)	4 (2–6)	− 0.622	0.534
HQER^h^ (%)	50 (33.33–70.83)	55.56 (33.33–71.43)	1.674	0.094
TQE^i^ (n)	0 (0–1)	0 (0–1)	− 0.827	0.408
TQER^j^ (%)	0 (0–9.09)	0 (0–9.09)	− 0.212	0.832
**Embryo transfer type**				
Cleavage-stage (n)	2 (2–2)	2 (2–2)	− 1.073	0.283
Blastocyst (n)	1 (1–1)	1 (1–2)	− 0.729	0.466
No. of embryo transferred (n)	2 (2–2)	2 (2–2)	− 0.804	0.421

^a^Body mass index.

^b^Gonadotropin.

^c^Progesterone.

^d^Human chorionic gonadotropin.

^e^In-vitro fertilization.

^f^Intracytoplasmic sperm injection.

^g^High-quality embryo.

^h^High-quality embryo rate.

^i^Top quality embryo.

^j^Top quality embryo rate.

* *P* < .05.

### Pregnancy outcome measures

As indicated in Table 3, the cancellation rates of fresh cycles and CLBRs were statistically significantly reduced in younger women with high BMIs compared to those with normal BMIs (28.6% vs. 34.8%, *P* < 0.001; 73.7% vs. 76.8%, *P* = 0.034, respectively), but no significant differences were observed in the older women subgroup (Supplementary Table S3). Reasons for fresh cycle cancellation included no available embryos (n = 166, 2.30%), a high risk of ovarian hyperstimulation syndrome (OHSS) (n = 1386, 19.17%), serum P ≥ 1.5 ng/ml on the hCG trigger day (n = 569, 7.87%), endometrial thickness of < 6 mm or > 16 mm (n = 33, 0.46%), and others (n = 281, 3.89%).

**Table 3:** Comparison of pregnancy outcomes among normal and high BMI^a^ groups (< 38 year).

Pregnancy outcomes	Normal BMI^a^	High BMI^a^	χ^2^	*P* value
**Fresh cycles**				
CPR^b^, n (%)	2274 (59.3%)	415 (59.5%)	0.004	0.949
OPR^c^, n (%)	2048 (53.4%)	374 (53.6%)	0.005	0.941
FLBR^d^, n (%)	1975 (51.5%)	360 (51.6%)	0.001	0.981
Ectopic pregnancy rate, n (%)	57 (1.5%)	8 (1.1%)	0.485	0.486
Biochemical pregnancy rate, n (%)	135 (3.9%)	16 (3.1%)	2.772	0.096
ESAR^e^, n (%)	169 (7.4%)	33 (8.0%)	0.137	0.712
LSAR^f^, n (%)	73 (3.2%)	14 (3.4%)	0.030	0.863
Singleton pregnancy rate, n (%)	1384 (60.9%)	238(57.3%)	1.809	0.179
Multiple pregnancy rate, n (%)	890 (39.1%)	177 (42.7%)	1.809	0.179
Cancellation rate, n (%)	2048 (34.8%)	279 (28.6%)	14.679	< 0.001*
Implantation rate, n (%)	3184 (42.7%)	594 (43.9%)	0.687	0.407
**Cumulative cycles**				
CLBR^g^, n (%)	4517 (76.8%)	720 (73.7%)	4.494	0.034*

^a^Body mass index.

^b^Clinical pregnancy rate.

^c^Ongoing pregnancy rate.

^d^Fresh live birth rate.

^e^Early spontaneous abortion rate.

^f^Late spontaneous abortion rate.

^g^Cumulative live birth rate.

* *P* <0. 05.

Other pregnancy outcomes are presented in Table 3 and Supplementary Table S3. No significant differences in implantation rate, CPR, ongoing pregnancy rate (OPR), FLBR, ectopic pregnancy rate, biochemical pregnancy rate, early spontaneous abortion rate (ESAR), and late spontaneous abortion rate (LSAR) of fresh cycles for both age subgroups between the two BMI groups were noted. Additionally, no significant differences were found in the rates of singleton pregnancy and multiple pregnancy.

### Obstetric complications, perinatal outcomes, and congenital defects

For the younger women, the details of perinatal outcomes resulting from fresh live births, such as gestational age, birth weight, neonatal asphyxia and infection, neonatal intensive care unit (NICU) admission, and early neonatal death, are presented in Supplementary Table S4. The results between the two BMI groups were not significantly different, with the exception of the rate of cesarean section, which was significantly higher in the high BMI group compared with the normal BMI group (90.8% vs. 83.1%, *P* < 0.001). In addition, obstetric complications, except for the rates of HDP and placenta previa, were largely similar between the two BMI categories. The incidences of HDP (4.4% vs. 1.5%, *P* < 0.001) and placenta previa (0.6% vs. 2.2%, *P* = 0.040) in the high BMI group were significantly higher and lower, respectively, than those in the normal BMI group.

The rates of cesarean section (92.8% vs. 87.1%, *P* < 0.001) and HDP (6.7% vs. 3.1%, *P* < 0.001) resulting from cumulative live births in younger women with high BMIs had similar trends to those of fresh live births (Supplementary Table S6). Moreover, the incidence of fetal macrosomia (4.6% vs. 2.8%, *P* = 0.002; Supplementary Table S6) and birth defects involving cleft lip and palate (0.4% vs. 0.1%, *P* = 0.030; Supplementary Table S9) resulting from cumulative live births in younger women with high BMIs was significantly higher than in those with normal BMIs. These results were not significantly different compared to those of fresh live births (Supplementary Tables S4 and S8).

In contrast, no significant differences were found in perinatal outcomes, obstetric complications, and congenital defects resulting from both fresh and cumulative live births in the older subgroup (Supplementary Table S5, S7, S8, and S9).

### Multivariate logistic regression analysis of BMI-related CLBR

As shown in Table 4, younger women with high BMIs had significantly decreased odds of CLBR compared with those with normal BMIs, with an adjusted OR (95% CI) of 0.809 (0.701–0.933). However, no statistical differences in the probability of CLBR were noted between the high BMI group and the normal BMI group for older women (Supplementary Table S10).

**Table 4:** Multivariate logistic regression analysis of BMI^a^-related CLBR^b^ (< 38 year).

Variable	β^c^	Wald^d^	*P* value	OR^e^ (95% CI^f^)
BMI^a^ group	− 0.212	8.504	0.004*	0.809 (0.701–0.933)
AFC^g^	0.013	6.317	0.012*	1.013 (1.003–1.023)
No. of oocyte retrieval	0.069	175.893	< 0.001*	1.071 (1.061–1.082)

^a^Body mass index.

^b^Cumulative live birth rate.

^c^Regression coefficient.

^d^Chi-square value.

^e^Odds ratio.

^f^Confidence interval.

^g^Antral follicle count.

*P < 0.05.

## Discussion

Despite previous studies reporting that elevated BMI can be associated with poor pregnancy outcomes of fresh IVF cycles, no significant effects of maternal BMI on implantation rate, CPR, OPR, and FLBR in all subgroups were observed in fresh cycles. However, in the subgroup < 38 years, cycle cancellation rates were significantly higher in the normal BMI group compared with the high BMI group (34.8% vs. 28.6%, *P* < 0.001). This was mostly due to the high risk of OHSS and elevated serum P, which was related to the development of multiple follicles caused by ovulation induction. According to clinical experience, they had a high chance of fresh pregnancy, but the transfer was cancelled because of inappropriate ovarian stimulation by exogenous Gn. Therefore, fresh cycle pregnancy outcomes in the normal BMI group may have been underestimated.

Unlike recent studies that have demonstrated that obesity was associated with spontaneous abortion^28^ and 1.45 times higher odds of early miscarriage compared with women with normal weights^29^, our study showed no statistical differences in the abortion rates of fresh cycles. Poor embryo quality appears to be the main cause of spontaneous abortion. However, in this study, there was no difference in the number of TQEs and HQEs, as they were given priority for fresh cycle transfer, which may be the reason for the nonstatistical difference in abortion rates. Elevated BMI was also notably associated with fewer mature oocytes and available embryos in younger women, but we did not observe a statistically significant decline in blastocyst formation rates, HQE rates, and TQE rates in overweight and obese individuals versus normal-weight controls, which was inconsistent with results from Ioanna et al.^11^.

As the most important research index of this study, CLBR was significantly lower among younger women with high BMIs compared with the normal BMI group. This may have been due to a reduced number of available embryos at cleavage and blastocyst stages in the high BMI group. Nevertheless, the decrease in the number of embryos was triggered by a reduction in the number of retrieved oocytes and mature oocytes, which was statistically different for the younger women in both BMI groups. Previous animal studies have indicated that obesity adversely affects oocyte maturation and embryonic development^30–34^, which could be due to direct oocyte damage^32,33,35^ and the indirect effects of dysfunctional systemic maternal endocrinology and metabolism, such as hypercholesterolemia, elevations in glucose, insulin, or free fatty acids, and changes in adipokines^30^. However, research evidence from human studies is limited. Leary et al. observed that human oocytes from overweight and obese women were smaller and less likely to complete fertilization using time-lapse systems^36^. Moreover, the resulting embryos were more likely to reach the morula stage, and the trophectoderm of blastocysts had fewer cells^36^. Additionally, donor oocyte research demonstrated that obesity did not affect IVF outcomes in women using donor oocytes^37^, that the adverse effects of elevated BMI on oocyte development rather than endometrial receptivity may be the major factor impairing pregnancy outcomes, and that this adverse effect was more pronounced in younger women. However, further research is needed to confirm and consolidate these ideas.

Bidirectional communication of oocytes with surrounding cells is critical for oocyte development, and several studies have attempted to evaluate the impact of obesity on these ovarian cells. High fat diet-mice exhibited increased anovulation and decreased fertilization rates in vivo, which was accompanied by remarkably increased apoptosis, endoplasmic reticulum stress, lipid accumulation, and mitochondrial dysfunction in granulosa and cumulus cells^38^. Abnormalities of granulosa cells caused by obesity may reduce estradiol production, which resulted in significantly lower peak estradiol levels among overweight and obese women of any age in the current study, despite the increased Gn dose given to the younger women. Thus, impaired granulosa cell function further deteriorates oocyte quality and developmental potential, ultimately reducing the number of mature oocytes and available embryos^39^, which are the keys to achieving cumulative live births.

Moreover, the long-term adverse effects of obesity are mainly reflected in the birth safety and health of the offspring. Maternal obesity has been associated with an increased risk of GDM, HDP, pre-eclampsia, PPH, caesarean delivery, macrosomia, and neonatal death^40,41^. Additionally, women with obesity are at increased risk of a range of structural abnormalities, including neural tube defects, spina bifida, cardiovascular abnormalities, septal abnormalities, cleft lip and palate, anorectal atrexia, hydrocephalus, and limb reduction abnormalities^42^. Furthermore, the risk of caesarean delivery was reported to have increased by 50% in overweight women and more than two-folds in obese women compared to women with normal BMIs^43^.

In the present study, however, only maternal HDP and fetal macrosomia were found to be more likely in younger women with high BMIs, and no significant difference were noted in terms of congenital defects between different BMI groups, with the exception of cleft lip and palate in the younger women. These findings which were not entirely consistent with previous studies^40–42^. In addition, the cesarean delivery rate in younger women with high BMIs was significantly higher than that in the normal BMI group, although the rates were also high in the normal BMI group (92.8% vs. 87.1%, *P* < 0.001). Several reasons may have led to the above phenomenon. Firstly, adverse obstetric complications and neonatal outcomes, including pre-eclampsia, PPH, neonatal asphyxia and infection, NICU admission, and early neonatal death may be partly reduced by the high rates of caesarean delivery. This largely related to an earlier family planning policy in China. Most Chinese couples choose to have only one child. Therefore, an elective cesarean section was considered a reliable delivery method that could avoid some of the uncertainties and risks associated with vaginal birth. Second, obstetric and neonatal risks, such as GDM, HDP, preterm birth, NICU admission, and neonatal death, increased with maternal overweight and increasing severity of obesity^44–46^_._ A large cohort study^47^ of 1.2 million liveborn, singleton infants also showed that the risk of major congenital malformations and several organ specific malformations progressively increased when the BMI and obesity increased. However, due to ethnic and regional differences, the proportion of obesity and morbid obesity in this study were relatively low. Third, as the birth defects data in our study were gathered through telephone follow-up, which ended one month after birth, and no more information has been collected since then. Therefore, many minor defects may have been overlooked, although the overall conclusion is less likely to be affected. Nevertheless, results indicated that fetuses of overweight and obese women were more likely to be exposed to adverse intrauterine environments, resulting in an increased incidence of fetal macrosomia and cesarean delivery. This, in turn, may impair obstetric safety and increase the risk of childhood and adult obesity.

Interestingly, our findings indicated no significant differences in pregnancy outcomes, obstetric and neonatal complications, and congenital defects between the two BMI groups among older women (≥ 38 year), whether they were undergoing fresh cycles or cumulative cycles. Therefore, we believe that age remains a major factor affecting IVF/ICSI outcomes, and obesity has a greater adverse impact on younger women compared with older women. As a result, we can provide clear guidance based on age boundaries, where younger women (< 38 year) with elevated BMIs may be encouraged to consider losing weight before pregnancy, but older women (≥ 38 year) may not have to achieve weight loss before consider pregnancy, as balancing the risks of age-related fertility decline are vital. Although many interventions related to weight management, including exercise, medication, diet, and bariatric surgery, have been explored to reduce the negative effects of overweight and obesity on IVF/ICSI outcomes^48,49^, the data are largely mixed, and there are few high-quality studies to guide clinicians. Therefore, further research is needed to determine which weight management interventions are beneficial for improving short- and long-term outcomes in younger women undergoing IVF/ICSI treatments.

Overall, this is a large-scale study to comprehensively assess the impact of elevated BMI on short- and long-term outcomes following one IVF/ICSI stimulation in a Chinese population. Unlike other studies, which have focused exclusively on pregnancy outcomes of fresh cycles as primary outcomes, the primary finding in our study was CLBR. CLBR is a more accurate indicator of the state of all oocytes deriving from a single IVF/ICSI stimulation, which lent credibility and persuasiveness to the results. In addition, to reduce the heterogeneity of the study, only the routine long protocol was included, and women with polycystic ovary syndrome, hypertension, and diabetes prior to IVF/ICSI were excluded. Nevertheless, there were a few limitations to this study. This study was a single-center study, and compared with the normal weight group (n = 6174, 85.41%), the proportion of overweight (n = 992, 13.72%) and obese (n = 63, 0.87%) participants included in the analysis was relatively low due to race- and region-restricted. Therefore, the overweight and obese groups were combined for statistical purposes and could not be separately analyzed. Although this study found adverse effects of elevated BMI during IVF/ICSI treatments, these results may not be generalizable to other racial and ethnic groups due to the low number of high BMI patients included and high cesarean section rates, a larger and more diverse dataset may provide additional insights into its impact. Moreover, due to the retrospective nature of the study, further randomized controlled trials are needed to validate the results.

In summary, overweight and obesity may impair CLBR in younger women by directly or indirectly impairing oocyte maturation and embryonic development. Maternal overweight and obesity were also closely associated with HDP and fetal macrosomia in younger women, which could increase birth risks and economic burdens. Therefore, weight loss management may improve pregnancy outcomes and safety among younger women with elevated BMIs. Further randomized controlled trials of weight management interventions prior to IVF/ICSI procedures in overweight and obese women are needed to determine whether short- and long-term adverse effects for both mothers and their offspring could be reduced.

## Methods

### Patients

We performed a retrospective study on women who were first treated at the Reproductive Medicine Center of Tongji Hospital from January 1, 2014 to December 31, 2017. This was a non-interventional, single-center cohort study of patients undergoing routine long gonadotropin-releasing hormone (GnRH) agonist protocol. Ethic approval was granted by the institutional review board of Tongji Hospital (reference no. 20201203), with written informed consent provided by participants. Patient information was anonymous without any identifier at the time of retrospective chart review, and all methods were carried out in accordance with the Declaration of Helsinki regulations.

BMI was expressed as the weight in kilograms divided by the square of the height in meters (kg/m^2^). Based on the World Health Organization’s BMI classification, all patients with normal (18.5–24.9 kg/m^2^) and high (≥ 25 kg/m^2^) BMI who had undergone their first fresh IVF/ICSI cycles were included in the analysis. On the other hand, women with polycystic ovary syndrome, uterine factors such as intrauterine adhesion and mediastinum uterus, hypertension, diabetes, and thyroid dysfunction were excluded. Furthermore, all cycles of half-ICSI fertilization, donor oocytes, egg freezing, and preimplantation genetic diagnoses/screenings were not included. A total of 7701 women were enrolled and were followed up through May 2020, until either the delivery of one live infant or the discontinuation of treatment. A total of 464 patients did not achieve live birth that had embryos frozen from the first stimulation, and 8 women had become pregnant. Ultimately, 7229 women completed the follow-up (Supplementary Fig. S1).

### Protocol for ovarian stimulation

Controlled ovarian stimulation was performed using the routine long GnRH agonist protocol. Oocytes were retrieved transvaginally 34–36 h after hCG administration, and fertilization was assessed 16–18 h after routine insemination or ICSI. Blastomere number and regularity, as well as the presence and volume of cytoplasmic fragmentations were assessed 24 and 48 h later, respectively. According to the protocol developed by Chinese legislation, a limit of two embryos were transferred on day 3 or 5 after oocyte retrieval. The remaining available embryos were cultured for blastocyst formation. All patients also received luteal support after embryo transfer, including intramuscular injections of progesterone in oil or vaginally administered micronized progesterone.

### Embryo grading

The presence of two clearly distinct pronuclei, along with two individualized or fragmented polar bodies, 20 h after IVF/ICSI was considered normal fertilization. Day 3 embryos with 7–9 equal-sized cells with a < 10% degree of fragmentation rate were defined as HQE. Additionally, day 3 embryos with eight equal size blastomeres and no cytoplasmic fragments that were derived from day 2 embryos with four equal size blastomeres and no cytoplasmic fragments were defined as TQE^50^.

### Pregnancy outcomes

The primary pregnancy outcome was the cumulative live birth, defined as at least one liveborn baby at ≥ 20 weeks’ gestation resulting from an IVF/ICSI-initiated cycle, including all fresh and subsequent frozen embryo transfer cycles, until one live birth occurred or all embryos were used.

Other pregnancy outcomes assessed in the study included implantation rate, CPR, OPR, FLBR, ectopic pregnancy rate, biochemical pregnancy rate, ESAR, LSAR, singleton pregnancy rate, and multiple pregnancy rate per fresh embryo transfer cycle. Implantation rate was determined based on the number of gestational sacs detected by ultrasound scan 5–7 weeks after embryo transfer divided by the number of embryos transferred. Moreover, clinical pregnancy was defined as the presence of a gestational sac with observed fetal heart rate by ultrasound five weeks after embryo transfer, while ongoing pregnancy was defined as a pregnancy with fetal heart activity detected by ultrasound after 12 weeks of gestation. Fresh live birth was defined as at least one liveborn baby at ≥ 20 weeks’ gestation after fresh embryo transfer. Additionally, early spontaneous abortion was defined as miscarriage occurring during the first trimester of pregnancy, while late spontaneous abortion was defined as miscarriage occurring after the first trimester of pregnancy. Finally, multiple pregnancy was defined as more than one gestational sac detected by ultrasound scan 5–7 weeks after embryo transfer.

### Obstetric complications, perinatal outcomes, and congenital defects

Obstetric and perinatal complications as well as congenital defects of deliveries from fresh cycles and cumulative cycles were followed up completely. Obstetric complications included in this study were HDP, GDM, pre-eclampsia, preterm premature rupture of membranes, placenta previa, polyhydramnios, oligohydramnios, PPH, placental abruption, and placenta accreta.

Perinatal outcomes included mode of delivery, gestational age, weight at birth, neonatal asphyxia and infection, NICU admission, and early neonatal death. In particular, very preterm birth and preterm birth were defined as a live birth or stillbirth occurring before 32 and 37 gestation weeks, respectively. Very low birth weight and low birth weight were also defined as a birth weight lower than 1500 g and 2500 g, respectively. Moreover, fetal macrosomia was defined as birth weight > 4000 g, and early neonatal death was defined as the death of a liveborn baby within seven days of birth.

Based on the classifications in 10th revision of the International Statistical Classification of Diseases Q codes (Q00-Q99)^51^, congenital malformations followed up in this study included cleft lip, cleft palate, and congenital malformations originating from the nervous, circulatory, digestive, urogenital, and musculoskeletal systems.

### Statistical analysis

Data were analyzed using SPSS 20.0 software. Normally distributed continuous data were presented as mean ± standard deviation, nonnormally distributed continuous data were expressed as median and quartile interval (M [P25–P75]), and categorical data were reported as the number of cases and frequency (%). Independent sample t-tests were used for intergroup comparisons of normally distributed continuous data, while nonparametric tests (rank-sum) were used for nonnormal distributions. Additionally, Chi-square tests (or Fisher’s exact tests, if appropriate) were used to determine the statistical significance between percentages for categorical data. A multivariate logistic regression model was used to determine odds ratios (ORs) and associated 95% confidence intervals (CIs) when comparing CLBR between two BMI groups. Significant different indictors in the baseline data and study factors that may affect CLBR were considered as adjustment factors. Statistical significance was set at *P* < 0.05.

## Supplementary Information

Supplementary Information.

## Data Availability

The datasets used and/or analyzed during the current study are available from the corresponding author on reasonable request.

## References

[1] Guh DP (2009). The incidence of co-morbidities related to obesity and overweight: A systematic review and meta-analysis. BMC Public Health.

[2] Fatini C, Cirillo M, Coccia ME (2018). Assisted reproductive technology, comorbidities, and cardiovascular risk: The experience of an Italian center. J. Womens Health (Larchmt).

[3] Cirillo M, Coccia ME, Fatini C (2020). Lifestyle and comorbidities: Do we take enough care of preconception health in assisted reproduction?. J. Family Reprod. Health.

[4] World Health Organization (WHO). *Obesity and overweight*. https://www.who.int/news-room/fact-sheets/detail/obesity-and-overweight (2022).

[5] Ng M (2014). Global, regional, and national prevalence of overweight and obesity in children and adults during 1980–2013: A systematic analysis for the Global Burden of Disease Study 2013. Lancet.

[6] Practice Committee of the American Society for Reproductive Medicine (2015). Obesity and reproduction: A committee opinion. Fertil. Steril..

[7] Santangeli L, Sattar N, Huda SS (2015). Impact of maternal obesity on perinatal and childhood outcomes. Best Pract. Res. Clin. Obstet. Gynaecol..

[8] Schliep KC (2015). Effect of male and female body mass index on pregnancy and live birth success after in vitro fertilization. Fertil. Steril..

[9] Friedler S (2017). Should high BMI be a reason for IVF treatment denial?. Gynecol. Endocrinol..

[10] Vilarino FL, Bianco B, Christofolini DM, Barbosa CP (2010). Impact of body mass index on in vitro fertilization outcomes. Rev. Bras. Ginecol. Obstet..

[11] Comstock IA, Kim S, Behr B, Lathi RB (2015). Increased body mass index negatively impacts blastocyst formation rate in normal responders undergoing in vitro fertilization. J. Assist. Reprod. Genet..

[12] Luke B (2011). The effect of increasing obesity on the response to and outcome of assisted reproductive technology: A national study. Fertil. Steril..

[13] Luke B (2011). Female obesity adversely affects assisted reproductive technology (ART) pregnancy and live birth rates. Hum. Reprod..

[14] Moragianni VA, Jones SM, Ryley DA (2012). The effect of body mass index on the outcomes of first assisted reproductive technology cycles. Fertil. Steril..

[15] Sarais V (2016). A comprehensive analysis of body mass index effect on in vitro fertilization outcomes. Nutrients.

[16] Shah DK, Missmer SA, Berry KF, Racowsky C, Ginsburg ES (2011). Effect of obesity on oocyte and embryo quality in women undergoing in vitro fertilization. Obstet. Gynecol..

[17] Bellver J (2010). Female obesity impairs in vitro fertilization outcome without affecting embryo quality. Fertil. Steril..

[18] Sermondade N (2019). Female obesity is negatively associated with live birth rate following IVF: A systematic review and meta-analysis. Hum. Reprod. Update.

[19] Kluge L (2019). Cumulative live birth rates after weight reduction in obese women scheduled for IVF: follow-up of a randomized controlled trial. Hum. Reprod. Open.

[20] Einarsson S (2017). Weight reduction intervention for obese infertile women prior to IVF: A randomized controlled trial. Hum. Reprod..

[21] Faddy MJ, Gosden RG, Gougeon A, Richardson SJ, Nelson JF (1992). Accelerated disappearance of ovarian follicles in mid-life: Implications for forecasting menopause. Hum. Reprod..

[22] Broekmans FJ, Kwee J, Hendriks DJ, Mol BW, Lambalk CB (2006). A systematic review of tests predicting ovarian reserve and IVF outcome. Hum. Reprod. Update.

[23] Drakopoulos P (2016). Conventional ovarian stimulation and single embryo transfer for IVF/ICSI. How many oocytes do we need to maximize cumulative live birth rates after utilization of all fresh and frozen embryos?. Hum. Reprod..

[24] Cimadomo D (2018). Impact of maternal age on oocyte and embryo competence. Front. Endocrinol..

[25] Capalbo A, Hoffmann E, Cimadomo D, Ubaldi F, Rienzi L (2017). Human female meiosis revised: New insights into the mechanisms of chromosome segregation and aneuploidies from advanced genomics and time-lapse imaging. Hum. Reprod. Update.

[26] Lebovitz O (2021). Embryonic development in relation to maternal age and conception probability. Reprod. Sci. (Thousand Oaks, Calif.).

[27] Mehari M (2020). Advanced maternal age pregnancy and its adverse obstetrical and perinatal outcomes in Ayder comprehensive specialized hospital, Northern Ethiopia, 2017: A comparative cross-sectional study. BMC Pregnancy Childbirth.

[28] Zhou Y (2019). Association of maternal obesity in early pregnancy with adverse pregnancy outcomes: A Chinese prospective cohort analysis. Obesity (Silver Spring).

[29] Ghimire PR, Akombi-Inyang BJ, Tannous C, Agho KE (2020). Association between obesity and miscarriage among women of reproductive age in Nepal. PLoS ONE.

[30] Purcell SH, Moley KH (2011). The impact of obesity on egg quality. J. Assist. Reprod. Genet..

[31] Wu LL, Norman RJ, Robker RL (2011). The impact of obesity on oocytes: Evidence for lipotoxicity mechanisms. Reprod. Fertil. Dev..

[32] Luzzo KM (2012). High fat diet induced developmental defects in the mouse: Oocyte meiotic aneuploidy and fetal growth retardation/brain defects. PLoS ONE.

[33] Jia Z (2018). Resveratrol reverses the adverse effects of a diet-induced obese murine model on oocyte quality and zona pellucida softening. Food Funct..

[34] Marei WFA (2020). Differential effects of high fat diet-induced obesity on oocyte mitochondrial functions in inbred and outbred mice. Sci. Rep..

[35] Jungheim ES (2010). Diet-induced obesity model: Abnormal oocytes and persistent growth abnormalities in the offspring. Endocrinology.

[36] Leary C, Leese HJ, Sturmey RG (2015). Human embryos from overweight and obese women display phenotypic and metabolic abnormalities. Hum. Reprod..

[37] Jungheim ES (2013). IVF outcomes in obese donor oocyte recipients: A systematic review and meta-analysis. Hum. Reprod..

[38] Wu LL (2010). High-fat diet causes lipotoxicity responses in cumulus-oocyte complexes and decreased fertilization rates. Endocrinology.

[39] He J (2021). Theaflavin 3, 3'-digallate delays ovarian aging by improving oocyte quality and regulating granulosa cell function. Oxid. Med. Cell. Longev..

[40] Marchi J, Berg M, Dencker A, Olander EK, Begley C (2015). Risks associated with obesity in pregnancy, for the mother and baby: A systematic review of reviews. Obes Rev.

[41] Poston L (2016). Preconceptional and maternal obesity: Epidemiology and health consequences. Lancet Diabetes Endocrinol.

[42] Stothard K, Tennant P, Bell R, Rankin J (2009). Maternal overweight and obesity and the risk of congenital anomalies: A systematic review and meta-analysis. JAMA.

[43] Poobalan A, Aucott L, Gurung T, Smith W, Bhattacharya S (2009). Obesity as an independent risk factor for elective and emergency caesarean delivery in nulliparous women–systematic review and meta-analysis of cohort studies. Obes. Rev.: Off. J. Int. Assoc. Study Obes..

[44] Aune D, Saugstad O, Henriksen T, Tonstad S (2014). Maternal body mass index and the risk of fetal death, stillbirth, and infant death: A systematic review and meta-analysis. JAMA.

[45] Kim SS (2016). Obstetric and neonatal risks among obese women without chronic disease. Obstet. Gynecol..

[46] Cnattingius S (2013). Maternal obesity and risk of preterm delivery. JAMA.

[47] Persson M (2017). Risk of major congenital malformations in relation to maternal overweight and obesity severity: Cohort study of 1.2 million singletons. BMJ (Clin. Res. ed.).

[48] Kort JD, Winget C, Kim SH, Lathi RB (2014). A retrospective cohort study to evaluate the impact of meaningful weight loss on fertility outcomes in an overweight population with infertility. Fertil. Steril..

[49] Liu L (2020). Effect of pregravid obesity on perinatal outcomes in singleton pregnancies following in vitro fertilization and the weight-loss goals to reduce the risks of poor pregnancy outcomes: A retrospective cohort study. PLoS ONE.

[50] Huang B (2016). Elevated progesterone levels on the day of oocyte maturation may affect top quality embryo IVF cycles. PLoS ONE.

[51] World Health Organization (WHO). *ICD-10 for Congenital malformations, deformations and chromosomal abnormalities (Q00-Q99)*. https://icd.who.int/browse10/2019/en#/XVII (2019).

