# Supplementary material for: Impact of elevated body mass index on cumulative live birth rate and obstetric safety in women undergoing assisted reproductive technology
Source: Sci Rep. 2022 Nov 7;12:18858. doi: 10.1038/s41598-022-23576-0 (PMC9640544; doi:10.1038/s41598-022-23576-0)
Supplement: Supplementary file 1 — Supplementary Information. [file 41598_2022_23576_MOESM1_ESM.pdf]

Title: Impact of elevated body mass index on cumulative live birth rate and obstetric safety in women undergoing assisted reproductive technology

Author names and affiliations:

Dan Hu[1], M.D.

Affiliation: [1]Reproductive Medicine Center, Tongji Hospital, Tongji Medical College, Huazhong University of Science and Technology

Postal address: 1095 JieFang Avenue, Wuhan 430030, People's Republic of China

Email address: danhu1217@163.com

Bo Huang[1], Ph.D.

Affiliation: [1]Reproductive Medicine Center, Tongji Hospital, Tongji Medical College, Huazhong University of Science and Technology

Postal address: 1095 JieFang Avenue, Wuhan 430030, People's Republic of China

Email address: hb@tjh.tjmu.edu.cn

Min Xiong[1], Ph.D.

Affiliation: [1]Reproductive Medicine Center, Tongji Hospital, Tongji Medical College, Huazhong University of Science and Technology

Postal address: 1095 JieFang Avenue, Wuhan 430030, People's Republic of China

Email address: karren126@126.com

Junning Yao[1], Ph.D.

Affiliation: [1]Reproductive Medicine Center, Tongji Hospital, Tongji Medical College, Huazhong University of Science and Technology

Postal address: 1095 JieFang Avenue, Wuhan 430030, People's Republic of China

Email address: junningyao@hotmail.com

Shulin Yang[1], Ph.D.

Affiliation: [1]Reproductive Medicine Center, Tongji Hospital, Tongji Medical College, Huazhong University of Science and Technology

Postal address: 1095 JieFang Avenue, Wuhan 430030, People's Republic of China

Email address: yangshulin.1990@163.com

Ruxing Wu[1], Ph.D.

Affiliation: [1]Reproductive Medicine Center, Tongji Hospital, Tongji Medical College, Huazhong University of Science and Technology

Postal address: 1095 JieFang Avenue, Wuhan 430030, People's Republic of China

Email address: ruxingwu@163.com

***Correspondence:** Hanwang Zhang and Yiqing Zhao

Hanwang Zhang[1]*, Ph.D.

Affiliation: [1]Reproductive Medicine Center, Tongji Hospital, Tongji Medical College, Huazhong University of Science and Technology

Postal address: 1095 JieFang Avenue, Wuhan 430030, People's Republic of China

Phone number: +8613986151967

Email address: hwzhang605@126.com

Yiqing Zhao[1]*, Ph.D.

Affiliation: [1]Reproductive Medicine Center, Tongji Hospital, Tongji Medical College, Huazhong University of Science and Technology

Postal address: 1095 JieFang Avenue, Wuhan 430030, People's Republic of China

Phone number: +8613995628596

Email address: Yiqing_Zhao99@hotmail.com

Supplementary Figure S1 Flowchart of data selection

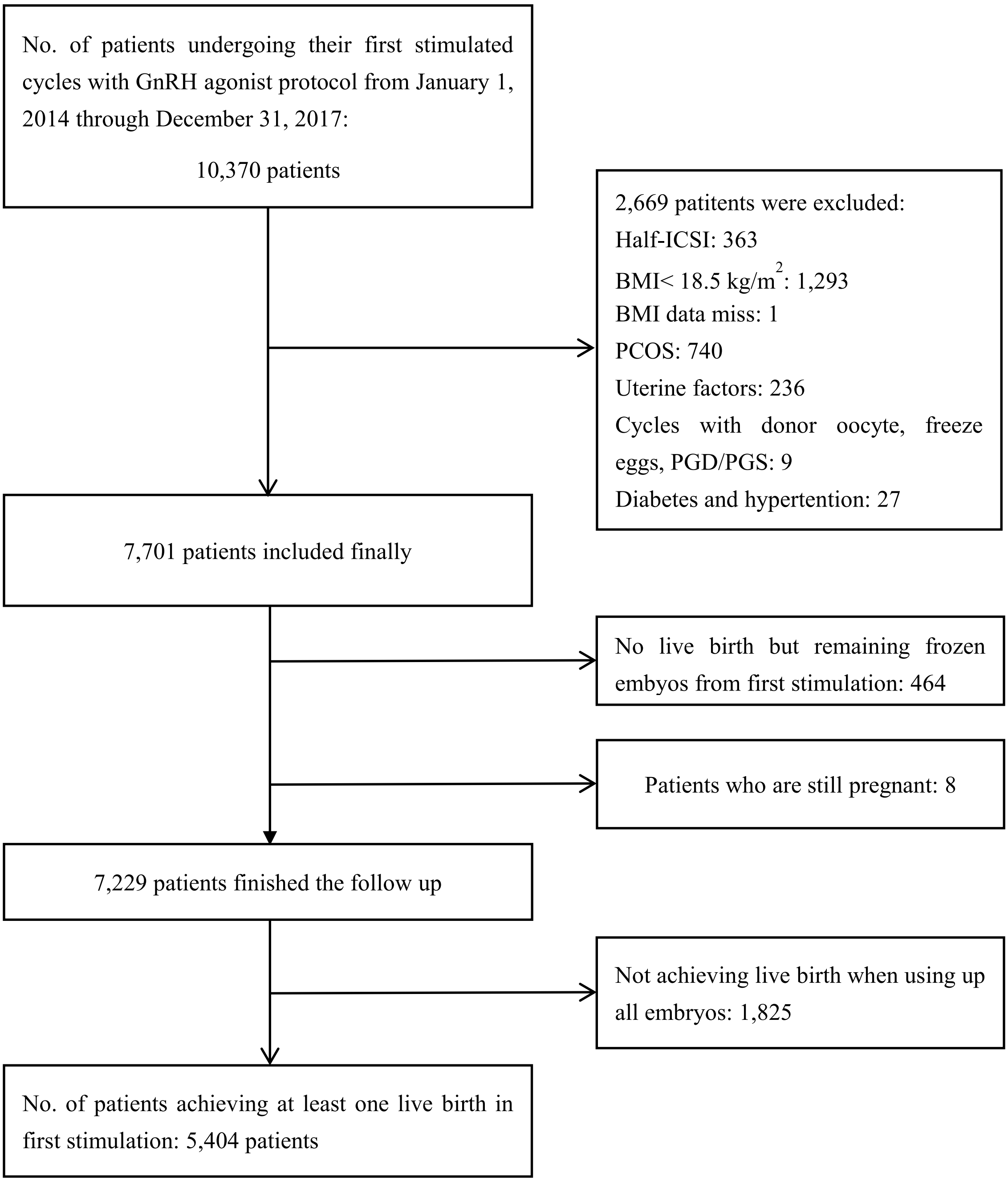

Supplementary Table S1: Comparison of baseline characteristics among normal and high BMI[a] groups (≥ 38 y)

	Normal BMI[a]	High BMI[a]	χ^2/z	*P* value
Infertility type			1.848	0.174
Primary	82 (28%)	28 (35.9%)		
Secondary	211 (72%)	50 (64.1%)		
Infertility etiology			2.945	0.567
Male	88 (30%)	25 (32.1%)		
Tubal	162 (55.3%)	45 (57.7%)		
DOR[b]	30 (10.2%)	7 (9%)		
EMT[c]	3 (1%)	1 (1.3%)		
Unexplained infertility	10 (3.4%)	0 (0%)		
Fertilization method			0.507	0.476
IVF[d]	229 (78.2%)	58 (74.4%)		
ICSI[e]	64 (21.8%)	20 (25.6%)		
Duration of infertility (years)	3 (2–8)	4 (2–10)	1.704	0.088
Basal FSH[f] (IU/L)	7.4 (6.37–8.41)	7.03 (6.2–7.81)	-1.850	0.064
AFC[g] (n)	11 (9–14)	12 (9–14)	0.887	0.375

[a]Body mass index
[b]Diminished ovarian reserve
[c]Endometriosis
[d]In-vitro fertilization
[e]Intracytoplasmic sperm injection
[f]Follicle-stimulating hormone
[g]Antral follicle count
* $P < .05$

Supplementary Table S2: Parameters of ovarian stimulation and embryos (≥ 38 y)

≥ 38y	Normal BMI[a]	High BMI[a]	z	*P* value
Duration of Gn[b] (days)	10 (9–11)	10 (9–11)	-2.802	0.005*
Total Gn[b] dose (IU)	2700 (2250–3187.5)	2625 (2325–3000)	-0.493	0.622
Peak estradiol (pg/ml)	3138 (2216–4891)	2796 (1954–3805)	-2.823	0.005*
Serum P[c] on hCG[d] day (ng/ml)	0.98 (0.74–1.27)	0.84 (0.63–1.13)	-2.358	0.018*
Endometrium thickness (mm)	11.4 (9.4–13.5)	11.2 (9–13.2)	-0.216	0.829
No. of follicles ≥ 14 mm (n)	9 (7–12)	9 (7–11)	-0.813	0.416
Retrieved oocytes (n)	10 (7–15)	10 (7–13)	-1.251	0.211
MII oocytes (n)	9 (7–13)	9 (6–12)	-1.512	0.131
MII oocytes rate (%)	94.44 (84.62–100)	93.54 (85.71–100)	-0.163	0.871
Insemination procedure (%)				
IVF[e]	66.67 (50–77.78)	66.67 (50–76.92)	-0.179	0.858
ICSI[f]	75 (63.39–85.71)	66.67 (52.27–85.16)	-1.199	0.230
Normally fertilized oocytes (n)	6 (4–9)	6 (4–8)	-1.872	0.061
Fertilization rate (%)				
IVF[e]	66.67 (50–77.78)	66.67 (50–75)	-0.577	0.564
ICSI[f]	75 (62.5–85.71)	66.67 (54.55–85.71)	-0.929	0.353
Cleavage (n)	7 (5–11)	7 (4–10)	-2.141	0.032*
Cleavage rate (%)	100 (100–100)	100 (100–100)	-0.639	0.523
Cleavage-stage embryos (n)	6 (4–9)	6 (4–8)	-1.094	0.274
Cleavage-stage embryos rate (%)	83.33 (71.43–100)	89.44 (75–100)	1.757	0.079

Blastocyst (n)	2 (1–4)	2 (1–3)	-0.790	0.430
Blastocyst formation rate (%)	50 (20–66.67)	50 (20–75)	0.222	0.824
Available blastocyst rate (%)	20 (0–40)	25 (0–50)	1.310	0.190
HQE[g] (n)	3 (1–5)	3 (1–5)	-0.354	0.723
HQER[h] (%)	50 (25–71.43)	52.27 (33.33–80)	1.108	0.268
TQE[i] (n)	0 (0–1)	0 (0–0)	-1.065	0.287
TQER[j] (%)	0 (0–8.33)	0 (0–0)	-0.924	0.356
Embryo transfer type				
Cleavage-stage (n)	2 (2–2)	2 (2–2)	-1.737	0.082
Blastocyst (n)	1 (1–1)	-	-	-
No. of embryo transferred (n)	2 (2–2)	2 (2–2)	-2.186	0.029*

[a]Body mass index
[b]Gonadotropin
[c]Progesterone
[d]Human chorionic gonadotropin
[e]In-vitro fertilization
[f]Intracytoplasmic sperm injection
[g]High-quality embryo
[h]High-quality embryo rate
[i]Top quality embryo
[j]Top quality embryo rate
* $P < .05$

Supplementary Table S3: Comparison of pregnancy outcomes among normal and high BMI[a] groups (≥ 38 y)

Pregnancy outcomes	Normal BMI[a]	High BMI[a]	χ^2	*P* value
**Fresh cycles**				
CPR[b], n (%)	79 (38.3%)	19 (33.3%)	0.481	0.488

OPR[c], n (%)	56 (27.2%)	16 (28.1%)	0.018	0.894
FLBR[d], n (%)	52 (25.2%)	15 (26.3%)	0.027	0.869
Ectopic pregnancy rate, n (%)	6 (2.9%)	1 (1.8%)	0.000	0.987
Biochemical pregnancy rate, n (%)	7 (3.4%)	1 (1.8%)	0.042	0.839
ESAR[e], n (%)	17 (21.5%)	2 (10.5%)	0.585	0.444
LSAR[f], n (%)	4 (5.1%)	1 (5.3%)	-	0.668
Single pregnancy rate, n (%)	65 (82.3%)	16 (84.2%)	0.000	1.000
Multiple pregnancy rate, n (%)	14 (17.7%)	3 (15.8%)	0.000	1.000
Cancellation rate, n (%)	87 (29.7%)	21 (26.9%)	0.229	0.632
Implantation rate, n (%)	93 (24.3%)	22 (19.6%)	1.046	0.306
**Cumulative cycles**				
CLBR[g], n (%)	131 (44.7%)	36 (46.2%)	0.052	0.820

[a]Body mass index
[b]Clinical pregnancy rate
[c]Ongoing pregnancy rate
[d]Fresh live birth rate
[e]Early spontaneous abortion rate
[f]Late spontaneous abortion rate
[g]Cumulative live birth rate

Supplementary Table S4: Perinatal outcomes and obstetric complications of fresh live births (< 38 y)

< 38 y	Normal BMI[a]	High BMI[a]	χ^2	*P* value
**Mode of delivery, n (%)**			13.638	<0.001*
Cesarean delivery	1642 (83.1%)	327 (90.8%)		
Vagina	333 (16.9%)	33 (9.2%)		

**Gestational weeks, n (%)**			0.040	0.980
Term (≥ 37 wk)	1550 (78.5%)	284 (78.9%)		
Preterm (< 37 wk)	390 (19.7%)	70 (19.4%)		
Very premature birth (< 32 wk)	35 (1.8%)	6 (1.7%)		
**Neonatal outcomes, n (%)**				
LBW[b] (< 2500 g)	685 (25.7%)	123 (25.0%)	0.097	0.756
VLBW[c] (< 1500 g)	36 (1.3%)	7 (1.4%)	0.017	0.896
Fetal macrosomia (> 4000 g)	56 (2.1%)	14 (2.8%)	1.072	0.301
Neonatal asphyxia	15 (0.6%)	1 (0.2%)	0.469	0.494
Neonatal infection	15 (0.6%)	3 (0.6%)	0.000	1.000
NICU[d] admission	231 (8.7%)	40 (8.1%)	0.146	0.702
Early neonatal death	17 (0.6%)	1 (0.2%)	0.720	0.396
**Obstetric complications, n (%)**				
HDP[e]	29 (1.5%)	16 (4.4%)	14.269	<0.001*
GDM[f]	54 (2.7%)	13 (3.6%)	0.840	0.359
Preeclampsia	0 (0%)	0 (0%)	-	-
PPROM[g]	38 (1.9%)	10 (2.8%)	1.102	0.294
Placenta previa	43 (2.2%)	2 (0.6%)	4.237	0.040*
Polyhydramnios	0 (0%)	0 (0%)	-	-
Oligohydramnios	0 (0%)	0 (0%)	-	-
PPH[h]	8 (0.4%)	2 (0.6%)	0.000	1.000

Placental abruption	0 (0%)	0 (0%)	-	-
Placenta accreta	0 (0%)	0 (0%)	-	-

[a]Body mass index
[b]Low birth weight
[c]Very low birth weight
[d]Neonatal intensive care unit
[e]Hypertensive disorders of pregnancy
[f]Gestational diabetes mellitus
[g]Preterm premature rupture of membranes
[h]Postpartum hemorrhage
* $P < .05$

Supplementary Table S5: Perinatal outcomes and obstetric complications of fresh live births (≥ 38 y)

≥ 38 y	Normal BMI[a]	High BMI[a]	χ^2	*P* value
**Mode of delivery, n (%)**			0.799	0.371
Cesarean delivery	48 (92.3%)	12 (80.0%)		
Vagina	4 (7.7%)	3 (20.0%)		
**Gestational weeks, n (%)**			3.555	0.169
Term (≥ 37 wk)	45 (86.5%)	11 (73.3%)		
Preterm (< 37 wk)	7 (13.5%)	3 (20.0%)		
Very premature birth (< 32 wk)	0 (0%)	1 (6.7%)		
**Neonatal outcomes, n (%)**				
LBW[b] (< 2500 g)	9 (15.3%)	2 (11.8%)	0.000	1.000
VLBW[c] (< 1500 g)	0 (0%)	0 (0%)	-	-
Fetal macrosomia (> 4000 g)	2 (3.4%)	0 (0%)	-	1.000
Neonatal asphyxia	0 (0%)	0 (0%)	-	-

Neonatal infection	0 (0%)	0 (0%)	-	-
NICU[d] admission	6 (10.2%)	1 (5.9%)	0.004	0.950
Early neonatal death	0 (0%)	0 (0%)	-	-
**Obstetric complications, n (%)**				
HDP[e]	1 (1.9%)	1 (6.7%)	-	0.400
GDM[f]	1 (1.9%)	1 (6.7%)	-	0.400
Preeclampsia	0 (0%)	0(0%)	-	-
PPROM[g]	0 (0%)	0 (0%)	-	-
Placenta previa	1 (1.9%)	0 (0%)	-	1.000
Polyhydramnios	0 (0%)	0 (0%)	-	-
Oligohydramnios	0 (0%)	0 (0%)	-	-
PPH[h]	0 (0%)	0 (0%)	-	-
Placental abruption	0 (0%)	0 (0%)	-	-
Placenta accreta	0 (0%)	0 (0%)	-	-

[a]Body mass index
[b]Low birth weight
[c]Very low birth weight
[d]Neonatal intensive care unit
[e]Hypertensive disorders of pregnancy
[f]Gestational diabetes mellitus
[g]Preterm premature rupture of membranes
[h]Postpartum hemorrhage

Supplementary Table S6: Perinatal outcomes and obstetric complications of cumulative live births (< 38 y)

< 38 y	Normal BMI[a]	High BMI[a]	χ^2	*P* value
**Mode of delivery, n (%)**			18.592	<0.001*

Cesarean delivery	3936 (87.1%)	668 (92.8%)		
Vagina	581 (12.9%)	52 (7.2%)		
**Gestational weeks, n (%)**			5.112	0.078
Term (≥ 37 wk)	3455 (76.5%)	529 (73.5%)		
Preterm (< 37 wk)	972 (21.5%)	169 (23.5%)		
Very premature birth (< 32 wk)	90 (2.0%)	22 (3.1%)		
**Neonatal outcomes, n (%)**				
LBW[b] (< 2500 g)	1314 (21.4%)	223 (22.5%)	0.637	0.425
VLBW[c] (< 1500 g)	73 (1.2%)	19 (1.9%)	3.569	0.059
Fetal macrosomia (> 4000 g)	173 (2.8%)	46 (4.6%)	9.576	0.002*
Neonatal asphyxia	30 (0.5%)	4 (0.4%)	0.012	0.913
Neonatal infection	41 (0.7%)	8 (0.8%)	0.246	0.620
NICU[d] admission	361 (5.9%)	68 (6.9%)	1.475	0.225
Early neonatal death	20 (0.3%)	1 (0.1%)	0.801	0.371
**Obstetric complications, n (%)**				
HDP[e]	141 (3.1%)	48 (6.7%)	22.436	<0.001*
GDM[f]	153 (3.4%)	31 (4.3%)	1.545	0.214
Preeclampsia	1 (0.0%)	0 (0%)	-	1.000
PPROM[g]	91 (2.0%)	21 (2.9%)	2.414	0.120
Placenta previa	96 (2.1%)	9 (1.3%)	2.422	0.120
Polyhydramnios	6 (0.1%)	1 (0.1%)	0.000	1.000
Oligohydramnios	2 (0.0%)	1 (0.1%)	-	0.358

PPH[h]	15 (0.3%)	2 (0.3%)	0.000	1.000
Placental abruption	3 (0.1%)	0 (0%)	-	1.000
Placenta accreta	2 (0.0%)	0 (0%)	-	1.000

[a]Body mass index
[b]Low birth weight
[c]Very low birth weight
[d]Neonatal intensive care unit
[e]Hypertensive disorders of pregnancy
[f]Gestational diabetes mellitus
[g]Preterm premature rupture of membranes
[h]Postpartum hemorrhage
* $P < .05$

Supplementary Table S7: Perinatal outcomes and obstetric complications of cumulative live births (≥ 38 y)

≥ 38 y	Normal BMI[a]	High BMI[a]	χ^2	*P* value
**Mode of delivery, n (%)**			0.000	1.000
Cesarean delivery	121 (92.4%)	33 (91.7%)		
Vagina	10 (7.6%)	3 (8.3%)		
**Gestational weeks, n (%)**			0.716	0.699
Term (≥ 37 wk)	103 (78.6%)	26 (72.2%)		
Preterm (< 37 wk)	26 (19.8%)	9 (25.0%)		
Very premature birth (< 32 wk)	2 (1.5%)	1 (2.8%)		
**Neonatal outcomes, n (%)**				
LBW[b] (< 2500 g)	30 (18.9%)	5 (12.2%)	1.005	0.316
VLBW[c] (< 1500 g)	3 (1.9%)	0 (0%)	-	1.000
Fetal macrosomia (> 4000 g)	6 (3.8%)	3 (7.3%)	0.306	0.580

Neonatal asphyxia	0 (0%)	0 (0%)	-	-
Neonatal infection	3 (1.9%)	0 (0%)	-	1.000
NICU[d] admission	11 (6.9%)	1 (2.4%)	0.501	0.479
Early neonatal death	0 (0%)	0 (0%)	-	-
**Obstetric complications, n (%)**				
HDP[e]	6 (4.6%)	2 (5.6%)	0.000	1.000
GDM[f]	5 (3.8%)	3 (8.3%)	0.467	0.494
Preeclampsia	0 (0%)	0 (0%)	-	-
PPROM[g]	1 (0.8%)	0 (0%)	-	1.000
Placenta previa	2 (1.5%)	0 (0%)	-	1.000
Polyhydramnios	0 (0%)	1 (2.8%)	-	0.216
Oligohydramnios	0 (0%)	0 (0%)	-	-
PPH[h]	1 (0.8%)	0 (0%)	-	1.000
Placental abruption	0 (0%)	0 (0%)	-	-
Placenta accreta	0 (0%)	0 (0%)	-	-

[a]Body mass index
[b]Low birth weight
[c]Very low birth weight
[d]Neonatal intensive care unit
[e]Hypertensive disorders of pregnancy
[f]Gestational diabetes mellitus
[g]Preterm premature rupture of membranes
[h]Postpartum hemorrhage

Supplementary Table S8: Congenital defects of fresh live births

Congenital defects	Normal BMI[a]	High BMI[a]	χ^2	*P* value
**< 38 y**				

Nervous system, n (%)	0 (0%)	0 (0%)	-	-
Circulatory system, n (%)	9 (0.3%)	4 (0.8%)	1.281	0.258
Cleft lip and cleft palate, n (%)	4 (0.1%)	1 (0.2%)	-	0.571
Digestive system, n (%)	3 (0.1%)	0 (0%)	-	1.000
Urogenital, n (%)	2 (0.1%)	0 (0%)	-	1.000
Musculoskeletal system, n (%)	6 (0.2%)	4 (0.8%)	2.883	0.090
**≥ 38 y**				
Nervous system, n (%)	0 (0%)	0 (0%)	-	-
Circulatory system, n (%)	0 (0%)	0 (0%)	-	-
Cleft lip and cleft palate, n (%)	0 (0%)	0 (0%)	-	-
Digestive system, n (%)	0 (0%)	0 (0%)	-	-
Urogenital, n (%)	0 (0%)	0 (0%)	-	-
Musculoskeletal system, n (%)	1 (1.7%)	0 (0%)	-	1.000

[a]Body mass index

Supplementary Table S9: Congenital defects of cumulative live births

Congenital defects	Normal BMI[a]	High BMI[a]	χ^2	*P* value
**< 38 y**				
Nervous system, n (%)	0 (0%)	0 (0%)	-	-
Circulatory system, n (%)	21 (0.3%)	7 (0.7%)	2.046	0.153
Cleft lip and cleft palate, n (%)	5 (0.1%)	4 (0.4%)	4.711	0.030*
Digestive system, n (%)	4 (0.1%)	0 (0%)	-	1.000
Urogenital, n (%)	7 (0.1%)	0 (0%)	0.266	0.606

Musculoskeletal system, n (%)	13 (0.2%)	5 (0.5%)	1.864	0.172
**≥ 38 y**				
Nervous system, n (%)	0 (0%)	0 (0%)	-	-
Circulatory system, n (%)	0 (0%)	0 (0%)	-	-
Cleft lip and cleft palate, n (%)	0 (0%)	0 (0%)	-	-
Digestive system, n (%)	0 (0%)	0 (0%)	-	-
Urogenital, n (%)	0 (0%)	0 (0%)	-	-
Musculoskeletal system, n (%)	1 (0.6%)	0 (0%)	-	1.000

[a]Body mass index
[*] *P* < .05

Supplementary Table S10: Multivariate logistic regression analysis of BMI[a]-related CLBR[b] (≥ 38 y)

Variable	β[c]	Wald[d]	*P* value	OR[e] (95% CI[f])
BMI[a] group	0.152	0.372	0.542	1.164 (0.715–1.895)

[a]Body mass index
[b]Cumulative live birth rate
[c]Regression coefficient
[d]Chi-square value
[e]Odds ratio
[f]Confidence interval
[*] *P* < .05
